# Biologic Use in Pediatric Patients With Hidradenitis Suppurativa: A Systematic Review

**DOI:** 10.1177/12034754211049711

**Published:** 2021-09-29

**Authors:** Muskaan Sachdeva, Patrick Kim, Asfandyar Mufti, Khalad Maliyar, Cathryn Sibbald, Afsaneh Alavi

**Affiliations:** 1 7938 Faculty of Medicine, University of Toronto, Canada; 2 12362 Michael G. DeGroote School of Medicine, McMaster University, Hamilton, Canada; 3 Division of Dermatology, Department of Medicine, University of Toronto, Canada; 4 7979 Section of Dermatology, Division of Pediatric Medicine, The Hospital for Sick Children, University of Toronto, Toronto, ON, Canada; 5 7985 Department of Dermatology, Mayo Clinic, Rochester, MN, USA

**Keywords:** Hidradenitis Suppurativa, acne inversa, treatment, biologic, pediatric, systematic review

## Abstract

**Background:**

There is currently at least 1 biologic (adalimumab) approved in North America for treatment of Hidradenitis Suppurativa in the pediatric population. However, no reviews or clinical trials have specifically analyzed the effectiveness and safety data of biologic use in this population. The objective of this systematic review is to identify and summarize the outcomes of biologic therapy in pediatric patients with HS.

**Methods:**

MEDLINE and EMBASE databases were used to conduct the search on Sept 18, 2020.

**Results:**

The 15 included studies consisted of 26 patients, with the mean age of 15 ± 2.3 years. Females accounted for 53.8% (*n* = 14/26) of cases. The mean duration of HS prior to biologic initiation was 3.5 ± 2.9 years, with the majority having Hurley Stage II. The 26 patients received 34 biologics in total: 85.3% treated with TNF alpha inhibitors (adalimumab *n* = 17, infliximab *n* = 10, etanercept *n* = 1, unspecified *n* = 1), 5.9% with IL-12/23 inhibitors (ustekinumab *n* = 2), 5.9% with IL-1 inhibitors (i.e., anakinra *n* = 2) and 2.9% received IL-23 inhibitors (i.e., guselkumab *n* = 1) biologics. Of the 26 patients, 23.1% (*n* = 6/26) experienced complete resolution (CR), 73.1% (*n* = 19/26) experienced partial resolution (PR), and 3.8% (*n* = 1/26) had no resolution outcomes reported. The time to resolution of HS lesions after biologic initiation ranged from 10 days to 11.5 months (mean: 5.1 months). No adverse events were reported in the studies.

**Conclusion:**

Although anti-TNF alpha were the most common biologics used for HS in pediatric cases, large-scale trials specific to pediatric patients with HS are needed to confirm these findings.

## Introduction

Hidradenitis Suppurativa (HS) is a chronic inflammatory condition affecting approximately 1% of the global population.^1,2^ It is characterized by painful nodules, abscesses, sinus tracts and scarring.^1^ HS is associated with an inflammatory response characterized by dysregulation of the immune system as a result of tumor necrosis factor (TNF)-α, interleukin (IL)−1β, IL-10, IL-23/*T* helper (Th) 17, and IL-12/Th1 pathways.^3,4^ Current treatment methods for HS, such as surgery, have not shown significant improvement over time.^5^ As such, the efficacy of biologic therapies are being explored, as promising results have been observed in biologic treatment for other inflammatory conditions including psoriasis and rheumatoid arthritis.^5^ Biologic therapies have recently been found effective at managing moderate-to-severe cases of hidradenitis suppurativa (HS).^6^

Adalimumab, a TNF-α inhibitor, is the only biologic approved by the Food and Drug Administration (FDA) and Health Canada for moderate-to-severe HS treatment.^7^ Studies to date in adults generally report highest efficacy with TNF-α inhibitors, specifically adalimumab and infliximab.^5,6,8^ Other biologics have shown variable results and require more data to define their efficacy and safety in individuals with HS.^8^

Although generally considered to have favorable side effect profiles, biologics have been associated with complications. Adverse events linked to biologic use have been reported in adult HS patients, including increased risk of infection, reactivation of latent tuberculosis and cancers.^5^ Additionally, lymphoma, and demyelinating disorders have been documented with biologic use in pediatric patients with psoriasis.^9^ However, little information is available on the effectiveness and safety of biologic use for HS management in pediatric population.^10^ This systematic review summarizes the outcomes of biologic therapies in pediatric HS and provides valuable information for dermatologists assessing the risks and benefits of different biologics for treating this population.

## Methods

This systematic review was conducted in accordance with the Preferred Reporting Items for Systematic Reviews and Meta-Analyses (PRISMA) guidelines.^11^

### Search Strategy

Searches were conducted using the EMBASE and MEDLINE in OVID on Sept 18, 2020. No language or date restrictions were applied. Variations of the following keywords were used for the search: “hidradenitis suppurativa,” “specific biologic,” and “children” (Supplemental File 1-2).

### Study Eligibility Criteria

Original articles were included in this systematic review if they (i) involved human participants, (ii) were observational (i.e., case reports, case series, cross-sectional or cohort studies) or experimental (i.e., randomized controlled trials) studies, (iii) involved biologics as an intervention, (iv) included pediatric patients with hidradenitis suppurativa, and (v) were written in the English language.

### Study Selection

Two reviewers (M.S and K.M.) independently screened titles, abstracts, and full texts of retrieved articles and determined study eligibility. Discrepancies or conflicts were resolved through discussion with a third reviewer (A.M.). Reference lists from all relevant articles were checked to identify additional studies not identified in the initial database search.

### Data Collection

Reviewers (M.S and P.K.) independently reviewed and extracted data from each study using a structured form. Conflicts were reviewed collectively and if consensus was not reached, a third reviewer was consulted (A.M.). Study design, patient demographic data, biologic treatments and resolution outcomes were extracted and summarized in Supplemental File 3. The following post-treatment outcomes were extracted and analyzed:

- Resolution:
  - Worsening: defined by exacerbation of HS lesions
  - No Improvement: defined by no changes in HS lesions
  - Partial Resolution: defined by improvement, yet lack of complete remission, of HS lesions
  - Complete Resolution: defined by total remission of HS lesions
- Resolution period: duration between onset and partial or complete resolution of HS lesions
- Recurrence of HS lesions
- Reported adverse events

### Level of Evidence Evaluation and Statistical Analysis

Level of evidence for all included articles was assessed independently by two reviewers (M.S. and P.K.) using the Oxford Centre for Evidence-Based Medicine 2011 Levels of Evidence.^12^ Due to the considerable heterogeneity of the included studies, a descriptive review was undertaken.

## Results

The search yielded 919 records after duplicates were removed. Following the title and abstract screening, 93 records were selected for a full-text review. In total, 15 studies met eligibility criteria and were used for data collection and analysis of 26 patients.^13-27^ The analysis of the level of evidence showed that 6 studies (40%) had a level of evidence of 4 and 9 studies (60%) had a level of evidence of 5. Overall, patients were between 6 and 17 years of age (mean: 15 years ± 2.3) at presentation and initiation of biologics. Females accounted for 53.8% (*n* = 14/26) of cases.

The average duration of HS prior to initiating biologics was 3.5 ± 2.9 years, with the majority presenting with Hurley Stage II disease. The 26 HS cases were treated with a total of 34 biologics: 85.3% received anti-TNF-α (adalimumab *n* = 17/34, infliximab *n* = 10/34, etanercept *n* = 1/34, and unspecified *n* = 1/34), 5.9% received anti-IL-12/23 (ustekinumab *n* = 2/34), 5.9% received anti-IL-1 (anakinra *n* = 2/34), and 2.9% received anti-IL-23 (guselkumab *n* = 1/34). Of the 26 patients, 23.1% (*n* = 6/26) experienced complete resolution (CR), 73.1% (*n* = 19/26) experienced partial resolution (PR), and 3.8% (*n* = 1/26) had no resolution outcomes reported. The time to resolution after biologic initiation ranged from 10 days to 11.5 months (mean: 5.1 months). Concomitant interventions were reported in 23.1% (*n* = 6/26) cases, majority (66.7%, *n* = 4/6) of which were antibiotics (Supplemental File 3).

Specifically, for anti-TNF-α, 15.4% (*n* = 4/26) achieved CR, and 61.5% (*n* = 16/26) achieved PR (Supplemental File 3). Mean CR period was reported in 3 cases to be 6.2 months, and relapse occurred in 10.7% (*n* = 3/26) of cases on anti-TNF-α. Three patients, all females aged 15-17, did not respond to adalimumab but thereafter had either PR or CR to anti-IL12/23 or anti-IL-23 blockade. The 2 cases treated with anti-IL-12/23 achieved CR within 11.3 months without relapse. PR was achieved with anti-IL-1, and anti-IL-23 also achieved PR at 6 months.

## Discussion

Our systematic review revealed that the majority of pediatric HS patients received biologics were treated with TNF-α inhibitors (85.3%). The most commonly used anti-TNF-α agents were adalimumab and infliximab, with only one patient treated with etanercept. Specifically, for anti-TNF-α, 15.4% of cases achieved CR, and 61.5% achieved PR. This complements the findings of another systematic review of both pediatric and adult cases treated with biologics, which documented highest efficacy with anti-TNF-α agents, adalimumab and infliximab, while etanercept was proven to be ineffective.^6^ Adalimumab was found to be more effective than infliximab.^6^ However, this study reviewed mostly adult patients and did not report pediatric data separately,^6^ whereas our review included only pediatric studies.^13-27^

In addition to anti-TNF-α , anti-IL-1 may be effective against HS as elevated TNF-α and IL-1β levels have been detected in HS lesions.^28^ IL‐1β and TNF-α levels in HS lesions were elevated 31‐fold and 5‐fold, compared to healthy skin.^28^ Furthermore, following follicular rupture, secondary bacterial colonization can result in an inflammatory cascade mediated by TNF-α.^29^ This may explain the mechanism by which TNF-α blockade leads to improvement in HS disease severity.^29^ However, the majority of the pathogenesis data is based on adult patients; further mechanistic studies specific to pediatric population are required to make conclusions.

Additionally, IL-12 and IL-23 expression has been found to be elevated in dermal macrophages in HS lesions, and both IL-12/T-helper cell (Th) 1 and IL-23/Th17 pathways can be activated by TLRs to initiate autoimmune responses.^30^ Laboratory evidence revealed that multiple TLR agonists alone can increase IL-23 expression, while several signals are required to enhance IL-12 production.^31^ IL-23 has an important role in recruiting and activating various inflammatory cells that induce chronic inflammation.^32^ Thus, therapeutics that selectively inhibit IL-12 and IL-23 may be effective in treating inflammatory immune-mediated diseases.^32^

Comorbidities are a common and important consideration in pediatric patients with HS.^33^ The associated comorbidities are reported in up to 85% of HS pediatric population, including obesity, metabolic syndrome, inflammatory bowel and joint disease, anxiety, and depression.^34,35^ It is unknown if earlier and more effective treatments impact comorbidities, but they are likely to mitigate the impact of HS on mental and physical health. The full impact of uncontrolled disease on mental and physical health is difficult to quantify in the long term. HS can also lead to significant socioeconomic impacts, highlighting the need for early and effective treatment.^35^

Nonbiologic treatments for HS include topical treatments (i.e., clindamycin, resorcinol, antiseptics), antibiotics (i.e., tetracycline, doxycycline, minocycline, rifampicin), and surgical interventions.^36^ While safe, topical treatments as solo therapy are largely ineffective in moderate to severe pediatric cases.^36^ Systemic antibiotics from the tetracycline family pose a risk for children under the age of 8 due to tooth discoloration and dental enamel hypoplasia.^36^ Other antibiotics are safer for children, including ertapenem, clindamycin, erythromycin, and metronidazole.^36^ However, the use of rifampicin in regions where tuberculosis is prevalent poses a risk due to antibiotic restsance.^36^ Surgical interventions are effective; however, they are invasive and are associated with recurrence and post-operative complications which include infection, scars, and wound separation.^36^ Deroofing interventions are another invasive, yet effective procedure in Hurley stage I and II pediatric patients and are associated with lower relapse rates and less post-operative complications compared to excisions.^37,38^ Although this is a surgical intervention, it can be done in office with local anesthesia and is well tolerated in older children.^37^ Additionally, it is important to note a newer train of thought that surgical treatment should be an adjunctive treatment that is done in tandem with biologic or medical therapy, as chronic lesions often persist despite being on biologic therapy.^36-38^ Based on our review, biologics are a promising noninvasive treatment method for HS, as they may help mitigate the risks associated with other treatment modalities. Our findings show a high efficacy for biologics, specifically TNF-α inhibitors. Furthermore, no adverse events were reported within the follow-up periods for the studies in this review.

Limitations of this systematic review include small sample size, observational nature of the included studies (i.e., case reports and series), and missing data on disease severity. The lack of larger trials and observational nature of the studies limits the scope of analysis and generalizability of our findings to all pediatric patients on biologic treatment. Additionally, due to the heterogeneity of the data, it may be difficult to attribute causality between improvement of HS outcomes and biologic use. Only one study reported Hidradenitis Suppurative Clinical Response (HiSCR), a standardized measure, which indicates at least a 50% reduction in total abscess and inflammatory nodule count relative to baseline.^17^ As a result, the varying measurement scores used in each case complicates the comparison between different biologics and the subsequent response to treatment. Also, studies show that differences in individuals’ response to therapy for HS also depend on genetic variations between patients, which may have confounded our results.^39^

Currently, adalimumab is indicated in Canada, the United States and Europe for adolescents aged 12-17 with HS, although the indication is based on clinical trials in adult population. To date, there are no North American guidelines for adolescent HS. Despite these limitations, we found that anti-TNF-α were the most common biologics used for pediatric HS. Further studies with larger sample sizes are required to confirm the findings reported in this systematic review.

## Supplemental Material

Online supplementary file 1 - Supplemental material for Biologic Use in Pediatric Patients With Hidradenitis Suppurativa: A Systematic Review Click here for additional data file. Supplemental material, Online supplementary file 1, for Biologic Use in Pediatric Patients With Hidradenitis Suppurativa: A Systematic Review by Muskaan Sachdeva, Patrick Kim, Asfandyar Mufti, Khalad Maliyar, Cathryn Sibbald and Afsaneh Alavi in Journal of Cutaneous Medicine and Surgery
