# Supplementary material for: Biologic Use in Pediatric Patients With Hidradenitis Suppurativa: A Systematic Review
Source: J Cutan Med Surg. 2021 Sep 29;26(2):176–80. doi: 10.1177/12034754211049711 (PMC8978460; doi:10.1177/12034754211049711)
Supplement: Online supplementary file 1 - Supplemental material for Biologic Use in Pediatric Patients With Hidradenitis Suppurativa: A Systematic Review [file sj-pdf-1-cms-10.1177_12034754211049711.pdf]

Supplemental Material

Biologic Use in Pediatric Patients with Hidradenitis Suppurativa: A Systematic Review

Muskaan Sachdeva (BHSc)[1], Patrick Kim (BHSc)[2], Asfandyar Mufti (MD)[3], Khalad Maliyar (BA)[1], Cathryn Sibbald (MD, FRCPC)[3,4], Afsaneh Alavi (MD, FRCPC)[1,5]

[1]Faculty of Medicine, University of Toronto, Canada

[2]Michael G. DeGroote School of Medicine, McMaster University, Hamilton, Canada

[3]Division of Dermatology, Department of Medicine, University of Toronto, Toronto, Canada

[4]Section of Dermatology, Division of Pediatric Medicine, The Hospital for Sick Children, University of Toronto, Toronto, Ontario, Canada

Database(s): **Ovid MEDLINE: Epub Ahead of Print, In-Process & Other Non-Indexed Citations, Ovid MEDLINE® Daily and Ovid MEDLINE®** 1946-Present**, Embase Classic+Embase** 1947 to 2020 September 17

Search Strategy:

#	Searches	Results
1	hidradenitis suppurativa.mp. [mp=ti, ab, ot, nm, hw, fx, kf, ox, px, rx, ui, an, sy, tn, dm, mf, dv, kw, dq]	6833
2	acne inversa.mp. [mp=ti, ab, ot, nm, hw, fx, kf, ox, px, rx, ui, an, sy, tn, dm, mf, dv, kw, dq]	935
3	verneuil's disease.mp. [mp=ti, ab, ot, nm, hw, fx, kf, ox, px, rx, ui, an, sy, tn, dm, mf, dv, kw, dq]	131
4	pyoderma fistulans significa.mp. [mp=ti, ab, ot, nm, hw, fx, kf, ox, px, rx, ui, an, sy, tn, dm, mf, dv, kw, dq]	11
5	TNF alpha.mp. [mp=ti, ab, ot, nm, hw, fx, kf, ox, px, rx, ui, an, sy, tn, dm, mf, dv, kw, dq]	304621
6	Etanercept.mp. [mp=ti, ab, ot, nm, hw, fx, kf, ox, px, rx, ui, an, sy, tn, dm, mf, dv, kw, dq]	41018
7	Golimumab.mp. [mp=ti, ab, ot, nm, hw, fx, kf, ox, px, rx, ui, an, sy, tn, dm, mf, dv, kw, dq]	8385
8	Infliximab.mp. [mp=ti, ab, ot, nm, hw, fx, kf, ox, px, rx, ui, an, sy, tn, dm, mf, dv, kw, dq]	67222
9	Rituximab.mp. [mp=ti, ab, ot, nm, hw, fx, kf, ox, px, rx, ui, an, sy, tn, dm, mf, dv, kw, dq]	108349
10	Anakinra.mp. [mp=ti, ab, ot, nm, hw, fx, kf, ox, px, rx, ui, an, sy, tn, dm, mf, dv, kw, dq]	7256
11	Dupilumab.mp. [mp=ti, ab, ot, nm, hw, fx, kf, ox, px, rx, ui, an, sy, tn, dm, mf, dv, kw, dq]	2848
12	Adalimumab.mp. [mp=ti, ab, ot, nm, hw, fx, kf, ox, px, rx, ui, an, sy, tn, dm, mf, dv, kw, dq]	43140
13	Ustekinumab.mp. [mp=ti, ab, ot, nm, hw, fx, kf, ox, px, rx, ui, an, sy, tn, dm, mf, dv, kw, dq]	9420
14	IL 12 23.mp. [mp=ti, ab, ot, nm, hw, fx, kf, ox, px, rx, ui, an, sy, tn, dm, mf, dv, kw, dq]	1073
15	IL 17.mp. [mp=ti, ab, ot, nm, hw, fx, kf, ox, px, rx, ui, an, sy, tn, dm, mf, dv, kw, dq]	45905
16	Brodalumab.mp. [mp=ti, ab, ot, nm, hw, fx, kf, ox, px, rx, ui, an, sy, tn, dm, mf, dv, kw, dq]	1435

17	Ixekizumab.mp. [mp=ti, ab, ot, nm, hw, fx, kf, ox, px, rx, ui, an, sy, tn, dm, mf, dv, kw, dq]	2370
18	Secukinumab.mp. [mp=ti, ab, ot, nm, hw, fx, kf, ox, px, rx, ui, an, sy, tn, dm, mf, dv, kw, dq]	4885
19	Guselkumab.mp. [mp=ti, ab, ot, nm, hw, fx, kf, ox, px, rx, ui, an, sy, tn, dm, mf, dv, kw, dq]	995
20	Risankizumab.mp. [mp=ti, ab, ot, nm, hw, fx, kf, ox, px, rx, ui, an, sy, tn, dm, mf, dv, kw, dq]	506
21	Tildrakizumab.mp. [mp=ti, ab, ot, nm, hw, fx, kf, ox, px, rx, ui, an, sy, tn, dm, mf, dv, kw, dq]	579
22	Certolizumab*.mp. [mp=ti, ab, ot, nm, hw, fx, kf, ox, px, rx, ui, an, sy, tn, dm, mf, dv, kw, dq]	8585
23	IL 1.mp. [mp=ti, ab, ot, nm, hw, fx, kf, ox, px, rx, ui, an, sy, tn, dm, mf, dv, kw, dq]	82430
24	CD20.mp. [mp=ti, ab, ot, nm, hw, fx, kf, ox, px, rx, ui, an, sy, tn, dm, mf, dv, kw, dq]	50452
25	IL 4.mp. [mp=ti, ab, ot, nm, hw, fx, kf, ox, px, rx, ui, an, sy, tn, dm, mf, dv, kw, dq]	104359
26	IL 23.mp. [mp=ti, ab, ot, nm, hw, fx, kf, ox, px, rx, ui, an, sy, tn, dm, mf, dv, kw, dq]	15012
27	Omalizumab.mp. [mp=ti, ab, ot, nm, hw, fx, kf, ox, px, rx, ui, an, sy, tn, dm, mf, dv, kw, dq]	11223
28	Mirikizumab.mp. [mp=ti, ab, ot, nm, hw, fx, kf, ox, px, rx, ui, an, sy, tn, dm, mf, dv, kw, dq]	118
29	Vedolizumab.mp. [mp=ti, ab, ot, nm, hw, fx, kf, ox, px, rx, ui, an, sy, tn, dm, mf, dv, kw, dq]	4935
30	Natalizumab.mp. [mp=ti, ab, ot, nm, hw, fx, kf, ox, px, rx, ui, an, sy, tn, dm, mf, dv, kw, dq]	13394
31	Canakinumab.mp. [mp=ti, ab, ot, nm, hw, fx, kf, ox, px, rx, ui, an, sy, tn, dm, mf, dv, kw, dq]	3919
32	Tocilizumab.mp. [mp=ti, ab, ot, nm, hw, fx, kf, ox, px, rx, ui, an, sy, tn, dm, mf, dv, kw, dq]	16797
33	Abatacept.mp. [mp=ti, ab, ot, nm, hw, fx, kf, ox, px, rx, ui, an, sy, tn, dm, mf, dv, kw, dq]	13269
34	children.mp. [mp=ti, ab, ot, nm, hw, fx, kf, ox, px, rx, ui, an, sy, tn, dm, mf, dv, kw, dq]	2502660
35	child*.mp. [mp=ti, ab, ot, nm, hw, fx, kf, ox, px, rx, ui, an, sy, tn, dm, mf, dv, kw, dq]	5474004
36	infant.mp. [mp=ti, ab, ot, nm, hw, fx, kf, ox, px, rx, ui, an, sy, tn, dm, mf, dv, kw, dq]	2052778
37	youth.mp. [mp=ti, ab, ot, nm, hw, fx, kf, ox, px, rx, ui, an, sy, tn, dm, mf, dv, kw, dq]	161709
38	adolescent.mp. [mp=ti, ab, ot, nm, hw, fx, kf, ox, px, rx, ui, an, sy, tn, dm, mf, dv, kw, dq]	3784517
39	teen*.mp. [mp=ti, ab, ot, nm, hw, fx, kf, ox, px, rx, ui, an, sy, tn, dm, mf, dv, kw, dq]	74636
40	juvenile.mp. [mp=ti, ab, ot, nm, hw, fx, kf, ox, px, rx, ui, an, sy, tn, dm, mf, dv, kw, dq]	241477
41	young*.mp. [mp=ti, ab, ot, nm, hw, fx, kf, ox, px, rx, ui, an, sy, tn, dm, mf, dv, kw, dq]	2722294
42	1 or 2 or 3 or 4	7062
43	5 or 6 or 7 or 8 or 9 or 10 or 11 or 12 or 13 or 14 or 15 or 16 or 17 or 18 or 19 or 20 or 21 or 22 or 23 or 24 or 25 or 26 or 27 or 28 or 29 or 30 or 31 or 32 or 33 or 42	749835
44	34 or 35 or 36 or 37 or 38 or 39 or 40 or 41	9753676
45	42 and 43 and 44	1253
46	remove duplicates from 45	919

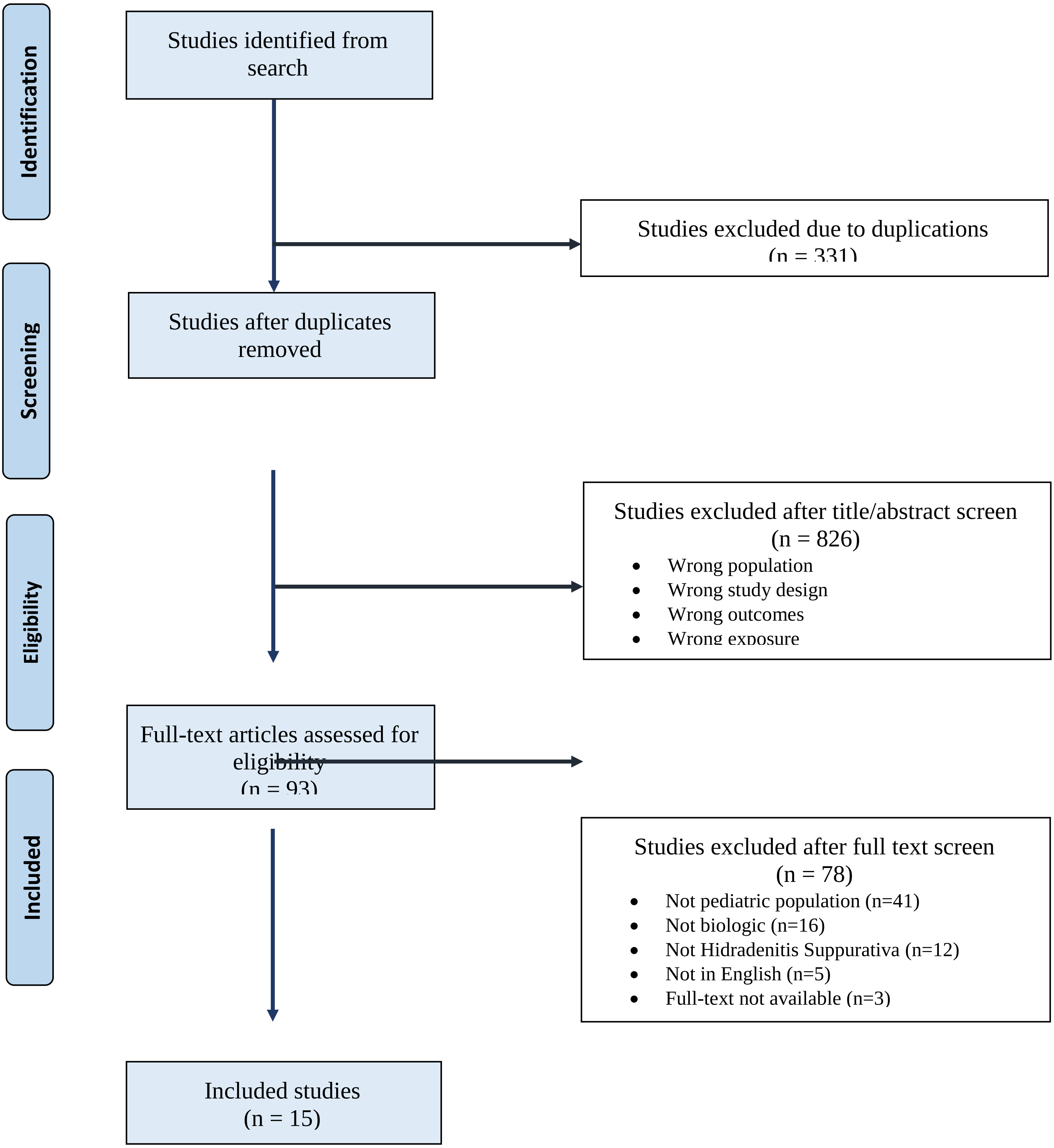

Supplemental File 2: Selection process for study inclusion.

Supplementary File 3: A Summary of Characteristics and Outcomes of Biologic Use in Pediatric Patients with Hidradenitis Suppurativa

Author, Year, Study Type	Level of Evidence	Age/ Sex	HS Duration (yrs)	Hurley Stage	Previous treatments	Location	Biologic (dose, frequency, duration)	Comorbidities and Risk Factors	Concomitant treatments	Resolution of HS Lesions (duration in months)	Relapse (duration in months)
Oranges, 2018, CR[15]	5	16/F	6	III	Antibiotics, estroprogestinics, isotretinoin, drainage procedures	Armpits , sternum, inguinal regions and submammary folds	Adalimumab (160 mg week 0, 80 mg at week 2, and then 40 mg every other week)	acne vulgaris, depression, bulimia, and pilonidal cyst	None	Partial (NR)	N
Genovese, 2019, CR[18]	5	15/M	0.67	NR	Isotretinoin	Neck and inguinal folds	Adalimumab (40 mg every 2 weeks, later every other week)	SAPHO syndrome (Synovitis, Acne, Pustulosis, Hyperostosis and Osteitis)	Oral prednisone 1.2 mg/kg/day, subcutaneous methotrexate 15mg/mq /week	Complete (6)	Y (NR)
Mahiques Santos, 2019, CR[21]	5	14/M	0	NR	Antibiotics, isotretinoin, systemic steroids	Face	Adalimumab (NR)	Facial acne	doxycycline	Partial (2)	N
Sahu, 2019, CR[22]	5	6/F	6	NR	Clindamycin and rifampicin (2 months), oral acitretin, oral isotretinoin (0.5 mg/kg/day increased to 1.0	Trunk and thighs	Adalimumab (40 mg initially and repeated after 4 weeks)	Moderate mental retardation, oligodontia, bilateral cataracts	None	Partial (2)	Y (3)

					mg/kg/day over 3 months)						
Adams, 2003, CR[13]	5	17/M	3	NR	Prednisone (5 mg twice daily for 2 years), cephalexin (500 mg twice daily for 2 weeks)	Thighs, scrotum, buttocks, inframammary folds	Infliximab (3 infusions 5 mg/kg at 0, 2, and every 6 weeks)	Pilonidal cyst, nodulocystic acne, ulcerative colitis, anemia, diabetes, obesity	None	Complete (6.5)	Y (5)
Ianhez, 2019, CR[19]	5	14/M	3	NR	Oral isotretinoin (0.75 mg/kg daily for 6 months)	Face, neck	Adalimumab (40 mg every other week for 4 months)	Keratitis Ichthyosis and Deafness (KID)'s syndrome, D50Y mutation	None	Partial (4)	NR
Lopes 2019, CR[20]	5	10/F	1	III	Topical clindamycin, oral clindamycin and rifampicin (2 months), oral prednisolone (20 mg twice)	perianal region	Adalimumab (160 mg, followed by 40 mg weekly)	None	None	Partial (6)	N
Provini, 2019, CR[25]	5	17/F	NR	NR	Topical steroids and mupirocin, oral clindamycin and rifampin (12 weeks), ciprofloxacin (2 weeks), oral prednisone (4 weeks),	Bilateral axillae, inframammary region, sternum, groin, and gluteal cleft	Biologic 1: Adalimumab (weekly for 6 months)	Depression, restrictive eating disorder	None	NR	N
							Biologic 2: Ustekinumab (90 mg every 8 weeks for 4 months, 90 mg every 4 weeks)		Spironolactone, doxycycline, Hyperbaric oxygen therapy	Complete (11.5)	
Monsalvez, 2019, CR[24]	5	17/F	4	NR	Conceptives, antiandrogens	NR	Biologic 1: Adalimumab (bowel disease dosage)	Ulcerative colitis	None	Complete (6)	N
							Biologic 2: Ustekinumab (NR)		Sulfone 50mg/day	Complete (11)	

Chinniah, 2014, CS[14]	4	16/F	NR	III	Isotretinoin, Systemic antibiotics, topical antibiotics	Axilla, submammary inguinal folds, abdomen	Biologic 1: Infliximab (infusion every 6 weeks, 9 doses)	acne conglobata, polycystic ovary syndrome, obesity(BMI=37)	None	Partial (11)	N
							Biologic 2: Adalimumab (80 mg week 0, 40 mg week 1 fortnightly for 6 months)		None	Complete (6)	N
Cusack, 2006, CS[16]	4	16/F	5	II	High dose oral antibiotics, dapsone, isotretinoin	Bilateral axillae, inguinal folds	Etanercept (loading dose followed by 25 mg twice per week subcutaneously)	Down's Syndrome	None	Partial (0.33 months or 10 days)	N
Fougerousse, 2020, CS[17]	4	15/M	1	II	Single-antibiotic therapy (n=4/12), combination antibiotic therapy (n=8/12), retinoids (n=3/12), extensive surgery (n=2/12)	Axillary, buttocks	Infliximab (5 mg/kg)	Dissecting cellulitis of the scalp, BMI 28.7	Antibiotics (n=1/12), sulfamethoxazole-trimethoprim (n=1/12)	Partial (4)	NR
		16/M	8	II		Axillary, inguinal, perineal, back of neck	Adalimumab (40 mg weekly)	Down syndrome, hypothyroidism, hypercholesterolemia, psoriasis, BMI 33.3		Partial (4)	NR
		16/M	1	II		Axillary, inguinal, scrotal	Infliximab (5 mg/kg)	Acne, BMI 41.5, family hx for psoriasis, ankylosing spondylarthritis		Partial (4)	NR
		16/F	1	III		Axillary, inguinal, perineal, submammary	Infliximab (10 mg/kg)	BMI 27.7		Partial (4)	NR
		15/F	2	II		Axillary, genital, follicular lesions	Adalimumab (40 mg weekly)	Ankylosing spondylarthritis, pilonidal cyst, acne conglobata, BMI 19.7, family hx for HS		Partial (4)	NR
		17/M	3	II		Axillary, inguinal	Adalimumab (40 mg weekly)	Acne, pilonidal cyst, BMI 29.1		Partial (4)	NR

		15/F	2	II		Axillary, inguinal, submammary	Adalimumab (40 mg weekly)	Acne, BMI 31.1, family hx for HS		Partial (4)	NR
		14/F	3	II		Axillary, inguinal	Infliximab (10 mg/kg)	Acne, pilonidal cyst, BMI 31.2, family hx for HS and Crohn's		Partial (4)	NR
		15/M	3	III		Axillary, buttocks, inguinal, perineal	Biologic 1: Adalimumab (40 mg weekly)	Acne, BMI 29.4		No improvement	NR
							Biologic 2: Infliximab (10 mg/kg)			Partial (4)	
		15/F	4	II		Axillary, inguinal, buttocks	Biologic 1: Infliximab (10 mg/kg)	Acne, BMI 30.2, family hx for HS		No improvement	NR
							Biologic 2: Adalimumab (40 mg weekly)			No improvement	
							Biologic 3: Anakinra (100 mg daily)			Partial (4)	
		17/M	3	II		Axillary, inguinal	Biologic 1: Adalimumab (40 mg weekly)	Acne, BMI 27.7		No improvement	NR
							Biologic 2: Infliximab (NR)			NR	
		16/M	13	II		Axillary, inguinal, follicular lesions	Infliximab (5 mg/kg)	Dissecting cellulitis of the scalp, acne, asthma, BMI 20.5		Partial (4)	NR
Kamal, 2016, RS[23]	4	16/M	NR	NR	Antibiotics, intralesional steroids, intralesional tacrolimus	axillae, buttocks, perianal	Anti-TNF alpha (NR)	Crohn's disease	None	Complete (NR)	NR
Casseres, 2019, RS[26]	4	15/F	NR	II	Spironolactone, isotretinoin, antibiotics	Axillae, groin	Biologic 1: Adalimumab	None	None	No improvement (3)	NR

							Biologic 2: Guselkumab (100mg at 0, 4 then every 8 weeks)		None	Partial (6)	
Hsiao, 2010, CS[27]	4	14/F	NR	NR	Minocycline, cyclosporine, tacrolimus ointment, 0.1%, clobetasol ointment, pulsed high-dose solumedrol, prednisone (NR); rifampin and clindamycin (NR)	Axilla, inframammary, gluteal cleft, inguinal folds, posterior neck, back folds, lower abdomen, thighs	Anakinra (NR)	Diabetes mellitus type 2, depression, arthritis, gastritis, chronic anemia, obesity, acanthosis nigricans, pyoderma gangrenosum	None	Partial (NR)	NR
CS: case series, CR: case report, BMI: body mass index, HS: hidradenitis suppurativa, NR: not reported, Y: yes, N: no<br>Partial response: improvement, but not complete resolution, of lesions<br>Complete response: total resolution of lesions											

Supplementary File 4: Summary of Outcomes for Each Biologic

Class	Biologic	Partial Resolution (n)	Complete Resolution (n)	No improvement (n)	Worsening (n)	Mean Resolution Period (months)	Relapse
**Anti-TNF**	Adalimumab	9	3	4	0	4.4	Yes: 2<br>No: 5
	Infliximab	7	1	1	0	5.2	Yes: 1<br>No: 1
	Etanercept	1	0	0	0	0.3	No
	NR	0	1	0	0	NR	NR
**IL-12/23**	Ustekinumab	0	2	0	0	11.3	No
**IL-23**	Guselkumab	1	0	0	0	6	NR
**IL-1**	Anakinra	2	0	0	0	4	NR

NR: not reported; n: number of patients
